# Supplementary figures and images for: MAPT Locus in Parkinson’s Disease Patients of Ashkenazi Origin: A Stratified Analysis
Source: Genes (Basel). 2023 Dec 28;15(1):46. doi: 10.3390/genes15010046 (PMC10815687; doi:10.3390/genes15010046)

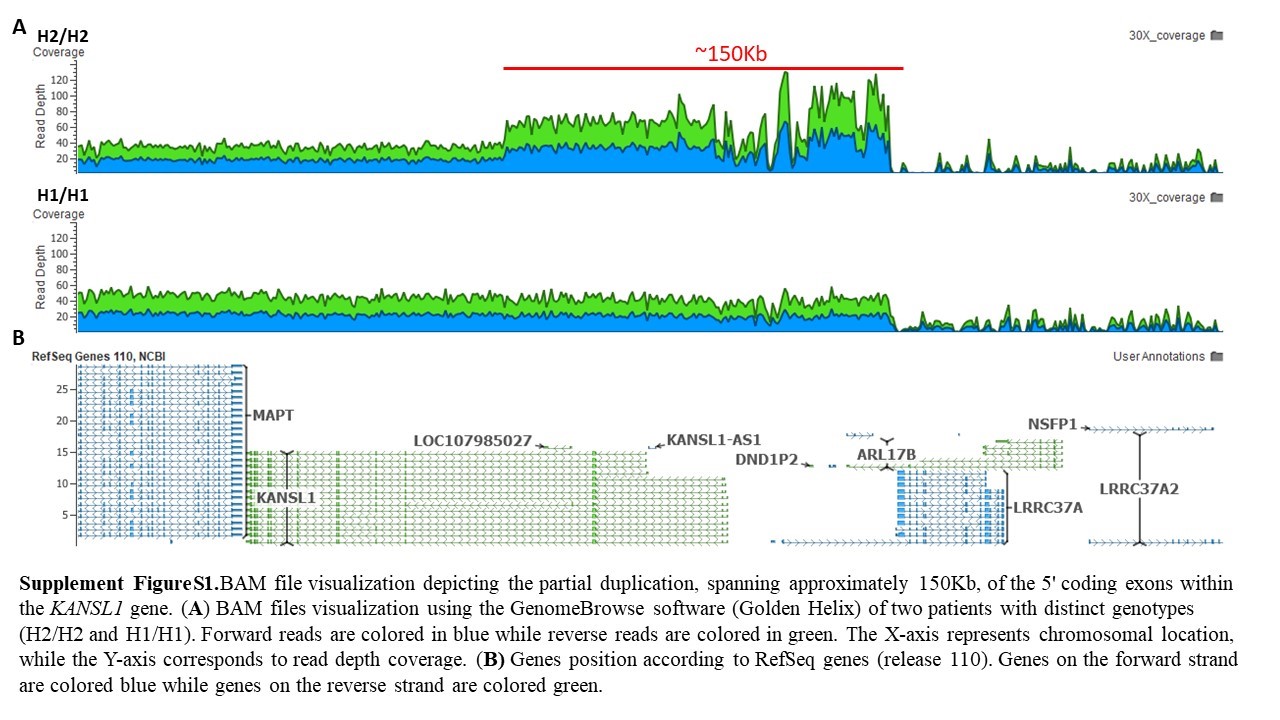

Supplement: Supplementary file 1 [file genes-15-00046-s001.zip › Shani_et_al_Supplement_Figure_S1_Nov_13_2023.jpg]

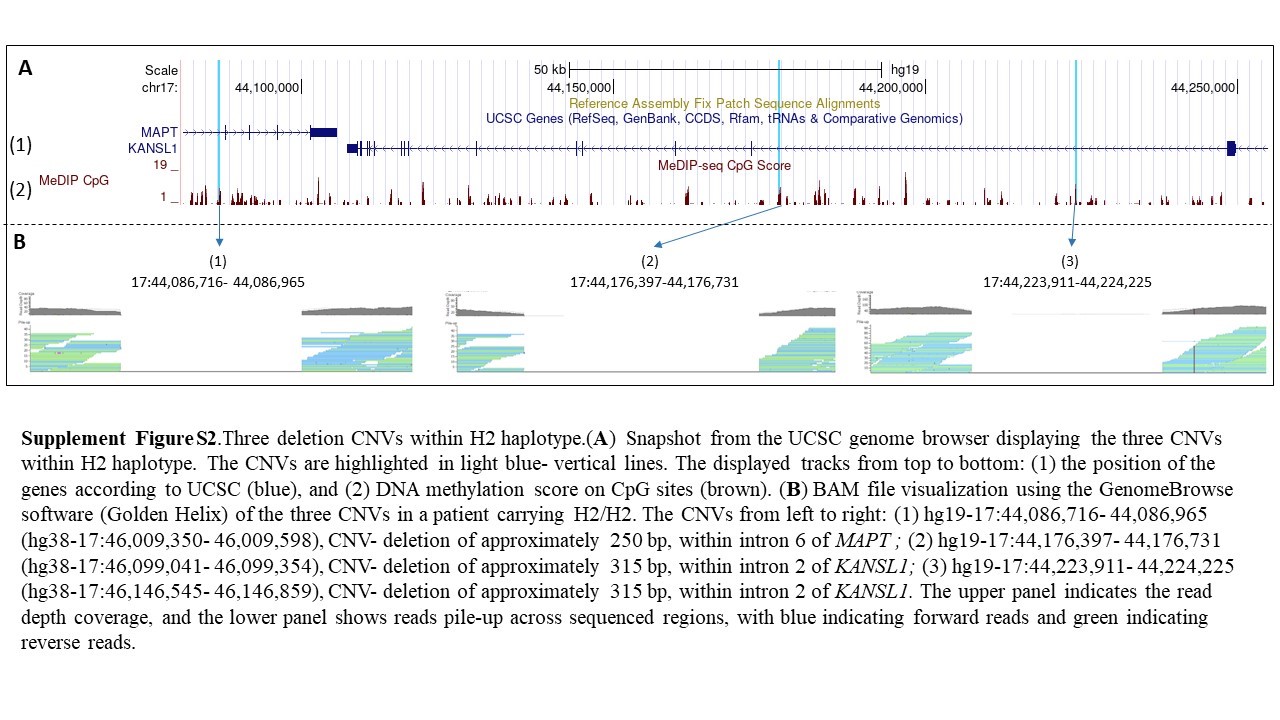

Supplement: Supplementary file 1 [file genes-15-00046-s001.zip › Shani_et_al_Supplement_Figure_S2_Nov_13_2023.jpg]
